# EEG-controlled tele-grasping for undefined objects

**DOI:** 10.3389/fnbot.2023.1293878

**Published:** 2023-12-19

**Authors:** Minki Kim, Myoung-Su Choi, Ga-Ram Jang, Ji-Hun Bae, Hyung-Soon Park

**Affiliations:** ^1^Department of Mechanical Engineering, Korea Advanced Institute of Science and Technology (KAIST), Daejeon, Republic of Korea; ^2^Applied Robot R&D Department, Korea Institute of Industrial Technology, Ansan, Republic of Korea

**Keywords:** brain-machine (computer) interface, telerobotics and teleoperation, human-robot collaboration, steady-state visual evoked potential (SSVEP), electroencephalogram (EEG)

## Abstract

This paper presents a teleoperation system of robot grasping for undefined objects based on a real-time EEG (Electroencephalography) measurement and shared autonomy. When grasping an undefined object in an unstructured environment, real-time human decision is necessary since fully autonomous grasping may not handle uncertain situations. The proposed system allows involvement of a wide range of human decisions throughout the entire grasping procedure, including 3D movement of the gripper, selecting proper grasping posture, and adjusting the amount of grip force. These multiple decision-making procedures of the human operator have been implemented with six flickering blocks for steady-state visually evoked potentials (SSVEP) by dividing the grasping task into predefined substeps. Each substep consists of approaching the object, selecting posture and grip force, grasping, transporting to the desired position, and releasing. The graphical user interface (GUI) displays the current substep and simple symbols beside each flickering block for quick understanding. The tele-grasping of various objects by using real-time human decisions of selecting among four possible postures and three levels of grip force has been demonstrated. This system can be adapted to other sequential EEG-controlled teleoperation tasks that require complex human decisions.

## 1 Introduction

Teleoperation enables the manipulation of a robotic device by a human operator distant from the robot's workspace (Niemeyer et al., 2016; Škulj et al., 2021). Traditional methods of robotic arm teleoperation focus on using the upper limb movements of humans including a joystick, haptic device, and inertial measurement unit (IMU) sensors (Zhao et al., 2017; Si et al., 2021; Škulj et al., 2021). Even though these methods enable precise control of various robotic arms in real-time, hands-free teleoperation can be useful in cases where human movement is limited which is already explained in the relevant literature. For instance, workers doing physical tasks with both hands in the industrial workspace would need the capability of hands-free teleoperation to conduct additional tasks simultaneously (Liu et al., 2021; Škulj et al., 2021). In addition to industrial purposes, it can be used to assist patients suffering from motor disabilities in an upper limb due to stroke or spinal cord injury (Meng et al., 2016; Chen et al., 2018; Quiles et al., 2022; Zhou et al., 2023). As grasping is an essential task to perform activities of daily living (ADL) (Roy et al., 2017), teleoperation of robotic grasping without upper limb motion can help these patients in fulfilling basic skills in everyday life.

Brain-Computer Interface (BCI) technology allows the teleoperation of robotic arms in the absence of physical movement through direct communication between the user's brain as a commander and the outside robot systems as an executor (Robinson et al., 2021). To record brain activity, invasive methods such as Electrocorticography (ECoG) provide highly accurate data with high spatial resolution but are less favored due to the risk of infection associated with the surgery (Meng et al., 2016; Xu B. et al., 2022). In contrast, Electroencephalography (EEG) is the most commonly used noninvasive method owing to its high temporal resolution, cost-effectiveness, simple use, and portability (Carpi et al., 2006; Aljalal et al., 2020). EEG inherently has lower accuracy and dimensionality compared to invasive methods (Meng et al., 2016). To address these limitations and cover a variety of commands from the EEG signals, the extraction of steady-state visually evoked potentials (SSVEP) from EEG has become a widely adopted approach in robotic arm control (Chen et al., 2019).

The SSVEP signals are resonance responses that can be observed in the event of providing a visual stimulus of flickering with a specified frequency, usually over 4 Hz (Diez et al., 2011). In existing studies of SSVEP-controlled robotic systems, the interface provided multiple flickering blocks of different frequencies linked to commands for the gripper motion. The human intention to move the robot system can be detected by identifying the gazed block from the extraction of the peak frequency in the SSVEP (Zhao et al., 2015; Qiu et al., 2016; Yang et al., 2017). In this work, we also used the peak frequency of the SSVEP to infer the human operator's desired command of motion through the EEG-BCI.

Several previous studies utilized the SSVEP-BCI to achieve EEG-controlled robotic arm systems and their grasping capabilities, as illustrated in Table 1. The subject could move the end-effector not only within a two-dimensional (2D) plane (Cao et al., 2021) but also in three-dimensional (3D) space (Chen et al., 2018; Zhu et al., 2020; Peng et al., 2022; Quiles et al., 2022; Zhou et al., 2023) by using six or more flickering blocks. The target object for grasping also could be selected by linking each flickering block to a placed object (Yang et al., 2017; Chen et al., 2019; Li and Kesavadas, 2022). Many SSVEP-BCI systems incorporated a shared control algorithm to tackle the difficulty of performing complex tasks using EEG signals. The shared control approach combines human decision-making with pre-programmed autonomous control for precise manipulation (Deng et al., 2020; Xu Y. et al., 2022). For instance, the fusion of computer vision and robotic autonomy enabled the grasping (picking) and releasing (placing) with enhanced task performance, requiring subjects only to issue onset commands or select objects (Yang et al., 2017; Cao et al., 2021; Zhou et al., 2023).

**Table 1:** Performance comparison table of previous EEG-controlled robotic arm systems.

**References**	**Task goal**	**Task difficulty**	**Human role**	**Performance**	
Cao et al. (2021)
Picking and placing five objects	Medium	2D movement, the onset of placing	Success rate: 85% (picking), 50% (placing)	Completion time: 53.50 s (picking), 97.20 s (placing)
Chen et al. (2019)
Picking and placing	Low	Object selection (among three)	Target selection in 6.5 s with 97.75 % of accuracy	
Chen et al. (2018)
Move-grasp-lift	High	3D movement, grasping	Total commands: 159.83 trials (five times of task)	Completion time: 639.33 s (five times of task)
Li and Kesavadas (2022)
Removing defective part	Low	Defective part selection	93.33 % accuracy for a 2.0 s time window	
Peng et al. (2022)
Reaching the designated position	Medium	3D movement	Total commands: 34.8 trials	Completion time: 174 s (for two tasks)
Zhu et al. (2020)
Grasp-lift-move	High	Grasping, lifting, and moving	92.09 % accuracy, Total commands: 68.73 trials	Completion time: 387.33 s
Yang et al. (2017)
Picking objects	Low	Object selection (among three)	Success rate: 90% (average of two subjects' results)	20 s to select a target object
Zhou et al. (2023)
Reach-grasp-drink	High	3D movement, the onset of grasping	Total commands: 9.40 (single object), 11.50 (three objects) trials	Completion time: 0.99 min (single object), 1.42 min (three objects)
Quiles et al. (2022)
Robotic arm control	Low	Axis rotation for 3D movement	Success rate: 71.5 %	around 3 min completion time
This work	Tele-grasping undefined object	High	3D movement, selecting posture and grip force, onset of grasping and releasing	Success rate: over 70% for all substep tasks	Total completion time: 115.3 s

Existing demonstrations of grasping, however, need further improvements to extend their utility beyond laboratory environments to the potential users of EEG-controlled teleoperation. In previous studies, the grasping task was accomplished by using pre-defined objects. The robot manipulator relied on object information through prior knowledge (training), and/or external sensors with computer vision for autonomous grasping. Human involvement was mostly limited to moving the gripper or selecting one of the pre-programmed motions. These pre-defined grasping situations could be done only by autonomous control without the need for human decision-making. On the other hand, in many demonstrations, robotic arm systems were situated close to the subject, facilitating the subject's observation of both the graphical user interface (GUI) and the robotic arm. This near positioning of the robot system restricted the grasping task to a structured environment. In a few studies where the human and robot were distant, the GUI only highlighted objects (Yang et al., 2017; Li and Kesavadas, 2022) or the video streaming of the robot workspace and the visual stimuli for the SSVEP were presented in different locations on the monitor (Cao et al., 2021). These setups constrained the involvement of the subject to specific parts of the task or potentially reduced the usability of the GUI.

In order to enable the utilization of the EEG-controlled grasping system by individuals with motor disabilities, the developed system has to be capable of grasping various objects based on their real-time intentions in even not pre-defined environments, to help ADLs. Objects used in everyday life vary widely in terms of their shape, fragility, weight, and size, and the object information is not pre-defined to the robot manipulator. The robot manipulator lacks the ability to determine appropriate posture and grip force without prior training (Billard and Kragic, 2019; Sun et al., 2021), whereas humans can readily determine the same even for unfamiliar objects (Si et al., 2021). So, it becomes possible to effectively grasp undefined objects within unstructured environments by affording the human operator the flexibility to select diverse grasping parameters. Accordingly, the provided GUI must encompass a wide range of decision options by utilizing shared control, all the while maintaining a user-friendly design.

Selecting various grasping parameters can be achieved by dividing the entire grasping task into multiple substeps and designing the GUI to facilitate teleoperation in a sequential procedure. Grasping can be divided into successive substeps of approaching the object, grasping it with an appropriate posture and grip force, and releasing it in the desired position (Lei et al., 2017; Newbury et al., 2023). Therefore, in this work the provided GUI allowed for human decision to select the posture and grip force, as well as translation of the gripper in the 3D space by the sequential procedure to grasp undefined objects in an unstructured task space.

**Figure 1:**
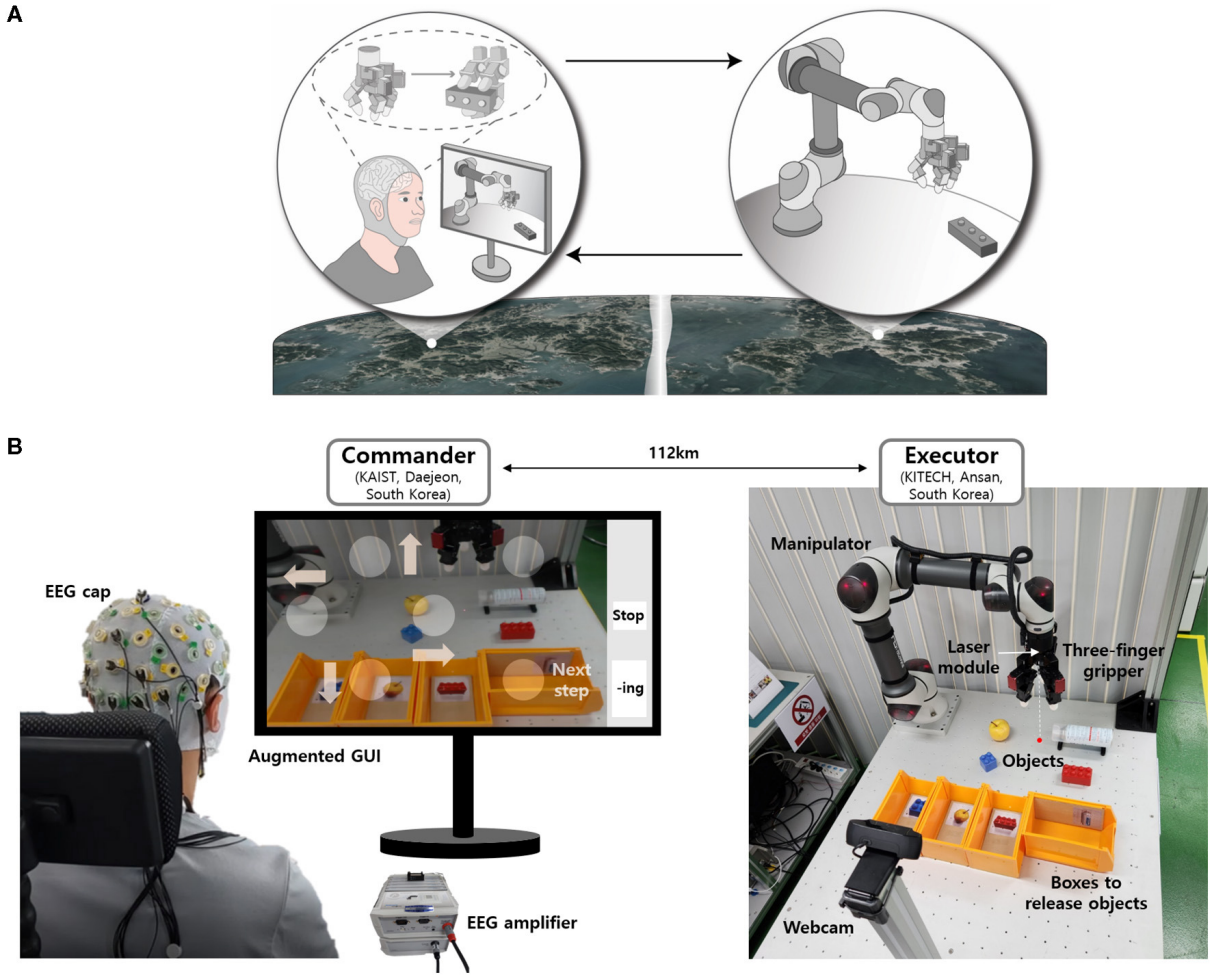
Illustration and realization of tele-grasping system based on real-time EEG and an intuitive GUI. **(A)** Conceptual schematic showing EEG-controlled tele-grasping. **(B)** Experiment setup for commander-executor tele-grasping.

In this work we developed a EEG-controlled teleoperation system that provides the human operator with various decision options for a tele-grasping task, as shown in Figure 1. We developed an intuitive GUI that can maximize the power of real-time human decision-making. The GUI was an augmented display of video streaming of the robotic system and flickering blocks with simple symbols of commands, updated for every substep. The tele-grasping of undefined objects was executed by involving the operator in all substeps for 3D movement of the gripper, selection of the grasping posture and grip force, and the onset of grasping and releasing. The shared control algorithm facilitated robust grasping based on the operator's command and the self-adjustment of the robot system.

## 2 System development

**Figure 2:**
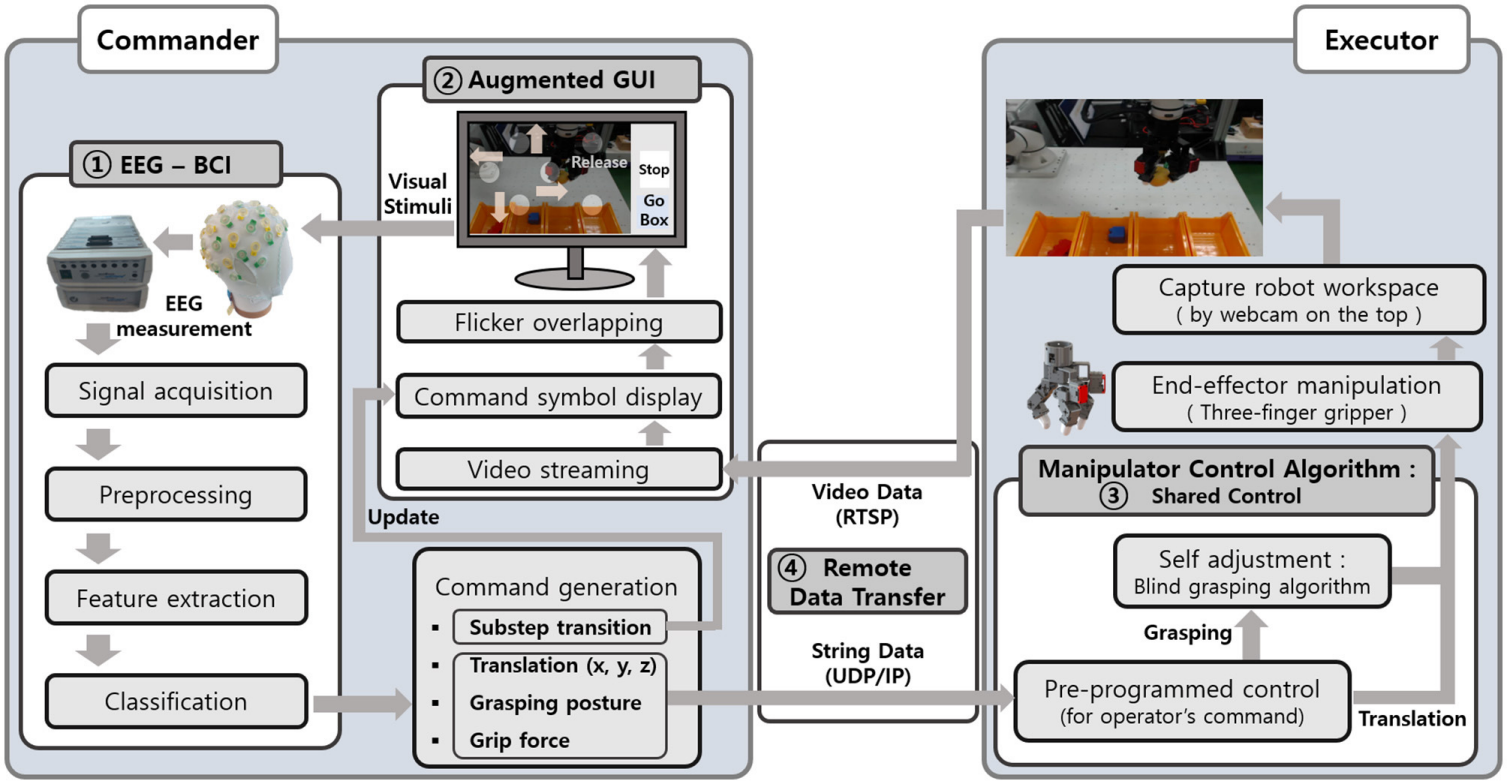
The schematic block diagram of the overall tele-grasping system.

Figures 1B, 2 show detailed illustrations describing the overall tele-grasping system. The system can be divided into four components: the EEG-BCI system, the augmented GUI, shared control algorithm, and remote data transfer, as categorized in Figure 2. The commander-executor configuration allowed operation over a distance of 100 km. At the commander location, the subject was provided with an augmented GUI that integrated video streaming of the executor and flickering blocks for SSVEP extraction. Upon fixating on a block for a predetermined duration, the EEG-BCI system generated a command, which was transmitted to the executor location. At the executor location, a robotic arm with a three-finger gripper was employed, and a webcam captured and transmitted the workspace in real-time. The shared control algorithm was utilized to manipulate the gripper based on the operator's command, with additional self-adjustment for stable grasping of the object. Various objects that were not pre-defined shapes or deformability were used, as shown in Figure 1B, and the human operator grasped and transported them to their associated boxes for release.

### 2.1 EEG-BCI system

The parietal and occipital cortex regions were chosen to measure EEG signals, as SSVEP is a response to external visual stimuli. Nine electrodes on these regions were selected for the extraction of SSVEP from the EEG measurement, specifically Pz, PO3, PO4, PO7, PO8, POz, O1, Oz, and O2, from the standard 64-channel actiCAP (EASYCAP GmbH, Germany), adhering to the international 10–20 EEG electrode placement standard. The actiCHamp system (Brain Products GmbH, Germany, ACBM16050605), and BrainVision Recorder (Brain Products GmbH) were used to amplify and record the signals. The sampling rate, *f*_*s*_ was downsampled to 250 Hz from the original rate of 500 Hz, and the raw signals were pre-processed using low- and high-pass filters with cutoff frequencies of 0.5 and 80 Hz, respectively, and a notch filter at 50 Hz.

The SSVEP response was elicited by presenting six circular blocks on a common LCD monitor (refresh rate of 64 Hz and screen resolution of 1,920 × 1,080) with distinct flickering frequencies of 5, 6, 7, 8, 9, and 11 Hz. The frequency of 10 Hz was excluded to avoid harmonic interference with 5 Hz. To extract the feature of the induced SSVEP signals, there have been developed several spatial filters to detect the peak frequency in the SSVEP. In this study, the standard canonical correlation analysis (CCA) method, which is famous for multichannel detection technique (Spüler et al., 2013; Kumar and Reddy, 2018), was applied to the segmented EEG data to obtain the peak frequency. The CCA method utilizes the projection vectors $W_{x}\in {\text{R}}^{N_{c}}$ and $W_{y}\in {\text{R}}^{2N_{h}}$ as the spatial filters by the linear transformation of the segmented EEG data $X\in {\text{R}}^{N_{c}\times N_{s}}$ and a set of sinusoidal harmonics of each flickering frequency (Equation 1) for the reference signals $Y_{k}\in {\text{R}}^{2N_{h}\times N_{s}}$, respectively. Then, the CCA method solves Equation (2) which seeks projection vectors *W*_*x*_ and *W*_*y*_ that maximize the correlation coefficient ρ between the pair of linear combinations of $W_{x}^{T}X$ and $W_{y}^{T}Y_{k}$ (Li and Kesavadas, 2022; Peng et al., 2022).

To enable the stable detection of the peak frequency in the application of the CCA method, we decided on the gaze duration as 4 s and introduced a preliminary calibration before the real-time tele-grasping task. So the pre-processed EEG signals were segmented into four-second windows in synchronization with the gazing duration *T*_*g*_, and then subjected to the CCA. The preliminary calibration of weighting the set of correlation coefficients, which algorithm will be elaborated in Section 3.1, was for the compensation of individual differences in the SSVEP response for each frequency between subjects.


(1) $$\begin{array}{l} \begin{array}{cc} Y_{k} & =\left[\begin{array}{c} \sin\left(2\pi f_{k}t\right)\\ \cos\left(2\pi f_{k}t\right)\\ .\\ .\\ .\\ \sin\left(2\pi N_{h}f_{k}t\right)\\ \cos\left(2\pi N_{h}f_{k}t\right) \end{array}\right] \end{array},t=\left[\begin{array}{c} \frac{1}{f_{s}},\frac{2}{f_{s}},...,\frac{N_{s}}{f_{s}} \end{array}\right] \end{array}$$

(2) $$\begin{array}{l} \begin{array}{cc} \max\rho_{k} & =\frac{E\left[W_{x}^{T}XY_{k}^{T}W_{y}\right]}{\sqrt{E\left[W_{x}^{T}XX^{T}W_{x}\right]E\left[W_{y}^{T}Y_{k}Y_{k}^{T}W_{y}\right]}},k=1,2,...,N, \end{array} \end{array}$$

In this study, *Y*_*k*_ is reference signals for the *k*th frequency *f*_*k*_ from *f* = [*f*_1_, ..., *f*_*N*_] = [5, 6, 7, 8, 9, 11], *N*_*c*_ = 9 is the number of channels, *N* = 6 is the number of flickering blocks with different frequencies, *N*_*h*_ = 5 is the number of harmonics, and *N*_*s*_ = *f*_*s*_*T*_*g*_ = 1000 is the number of sampling points. The frequency that gives the maximum correlation coefficient value is considered as the gazed block, as shown in Equation (3) :


(3) $$\begin{array}{l} f_{g}=\arg\max\rho_{k}, \end{array}$$

where *f*_*g*_ is the flickering frequency of the expected gazed block. The EEG-BCI system applied this CCA method for every four-second gazing duration is completed. As soon as the CCA method gave the result of the gazed block, the system immediately sent the corresponding command to the robot system, as summarized in the EEG-BCI box in Figure 2.

### 2.2 Augmented GUI design

**Figure 3:**
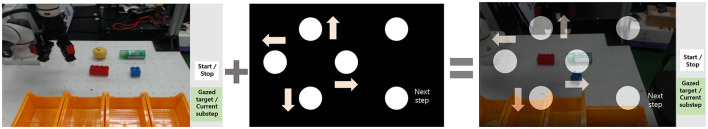
Augmented graphical user interface (GUI).

An augmented GUI was utilized to facilitate communication between the human operator and the robot manipulator. The term “augmented” denotes the integration of both visual stimuli for SSVEP and real-time video streaming of the robot system into a single display, as illustrated in Figure 3. Two separate windows for visual stimuli and video streaming were superimposed and displayed simultaneously by adjusting the transparency of the streamed image. This overlapping of windows aided in maintaining a consistent flickering with a specified frequency, which has a significant impact on the quality of SSVEP signal. Furthermore, it enhanced the user experience during tele-grasping by eliminating the need for the subject to switch between monitors.

**Figure 4:**
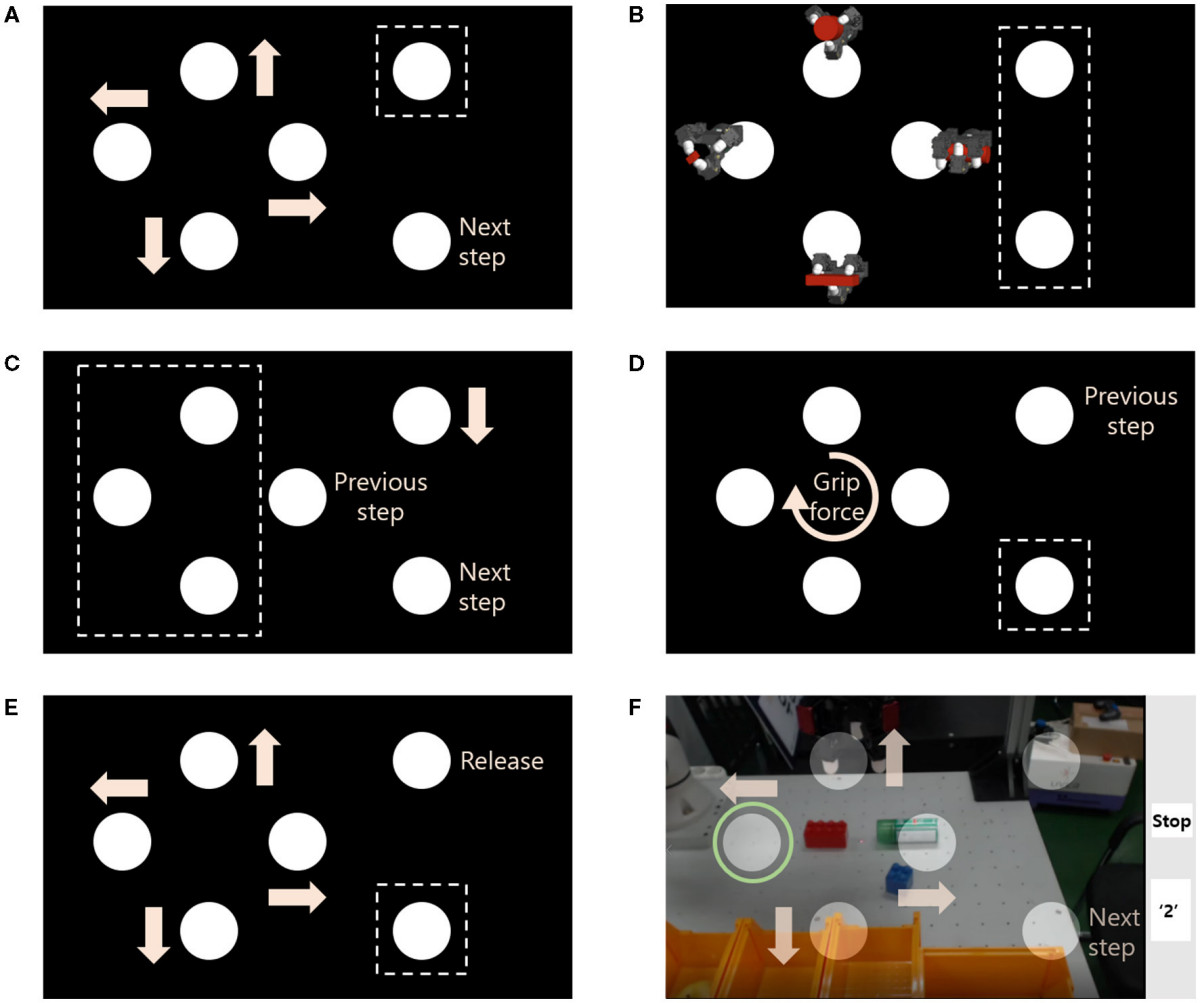
Symbolic display on the augmented GUI for each substep [Dotted squares indicate the invalid flicker(s)]. **(A)** 2D translation; **(B)** Selecting grasping posture; **(C)** Reaching an object; **(D)** Selecting amount of grip force; **(E)** Reaching a box; **(F)** Example snapshot of augmented GUI during the 2D translation.

For each substep the flickering blocks were assigned a different task differentiated by appropriate symbols adjacent to it (Figure 4). The visual stimuli window was designed to be independent of each substep, while symbols added in the window indicated the command associated with each block. For tele-grasping an undefined object by the human operator, the GUI had to accommodate the movement of the gripper in 3D space, selection of grasping posture, amount of grip force, and timing of grasp and release.

In the translation substep, four blocks corresponded to plane movement, with arrows used to symbolize each direction (Figures 4A, E). The same four blocks were used for selecting the grasping posture (Figure 4B) and grip force (Figure 4D), with images of the four postures next to each block and a clockwise arrow in the center of the four blocks to indicate the increasing level of the grip force, respectively. To reach an object, the rightmost upper block was used to descend the gripper, indicated by a downward arrow (Figure 4C). The transition between adjacent substeps was done by gazing at the designated block with phrases “Next step” or “Previous step.” Each substep had invalid blocks that were not allocated to specific commands, as illustrated by dotted rectangles in Figure 4.

The expected gazed block was displayed on the GUI to notify the subject of the progress of teleoperation. The EEG-BCI system transformed the gazed block into the designated command by applying the CCA method immediately after the four-second gazing duration. The human operator could not easily find the updated command and designated motion of the gripper. In order to confirm the selection, the gazed flickering block was highlighted by a green circular border on the GUI (Figure 4F). So the subject could not only directly understand the updated command, but also fix the erroneous situation even when the EEG-BCI system gave a wrong expectation of the gazed block.

In the lower right corner, a rectangular indicator was presented to provide information on the current substep and timing of gazing duration. This indicator was designed to differentiate between substeps by utilizing various background colors and captions. The gazing trial began with a caption of “on,” followed by “-ing” during the gazing period, and an assigned number of the gazed block at the end of the trial. For example, in Figure 4F, a white background represented the 2D movement substep, and the number “2” indicated the conclusion of the gazing and the leftward gripper movement. Other substeps, such as reaching an object, grip force selection, and reaching a box were indicated using red, blue, and yellow backgrounds, and were labeled as “down,” “grasp,” and “go to box.”

### 2.3 Shared control of the robot system

#### 2.3.1 The robot hardware for the executor

**Figure 5:**
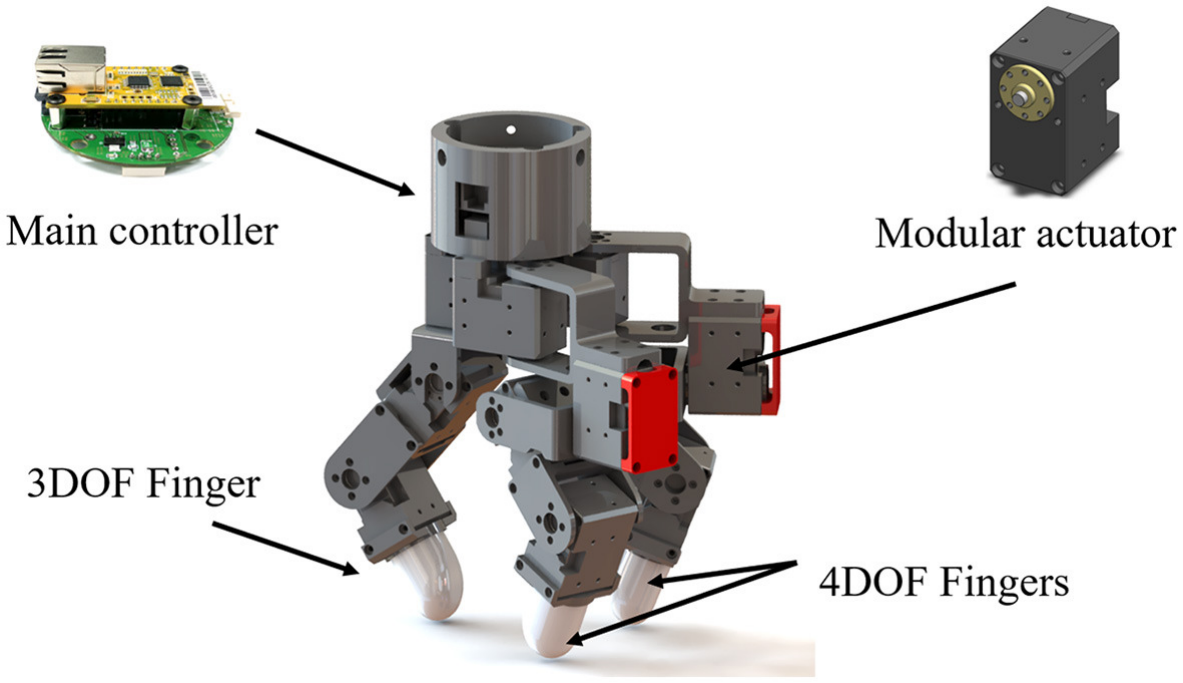
Three-finger gripper used in the tele-grasping.

The executor robotic system comprised the manipulator RB5-850 (Rainbow Robotics, Korea) and an independently developed gripper for the end-effector operation. The robotic arm had 6 degrees of freedom (DOF) from the root to the end-effector, while the three-finger gripper possessed a total of 11 DOF as shown in Figure 5. The gripper's initial position was fixed at the center of the table, and the orientation of the gripper was fixed downwards, as all objects were placed on a table (Figure 1B). The initial height was approximately 20 cm from the table. In addition, a practical laser pointer placed at the center of the gripper illuminated its position in the horizontal plane. During object manipulation of the gripper, the robot manipulator remained stationary. The gripper was capable of moving within the workspace of 50 × 50 × 40 (cm) and was programmed to move 5 or 10 cm per command along the Cartesian coordinate system per command to reach the object and transport it to the box, respectively.

#### 2.3.2 Grasping postures and grip force

**Figure 6:**
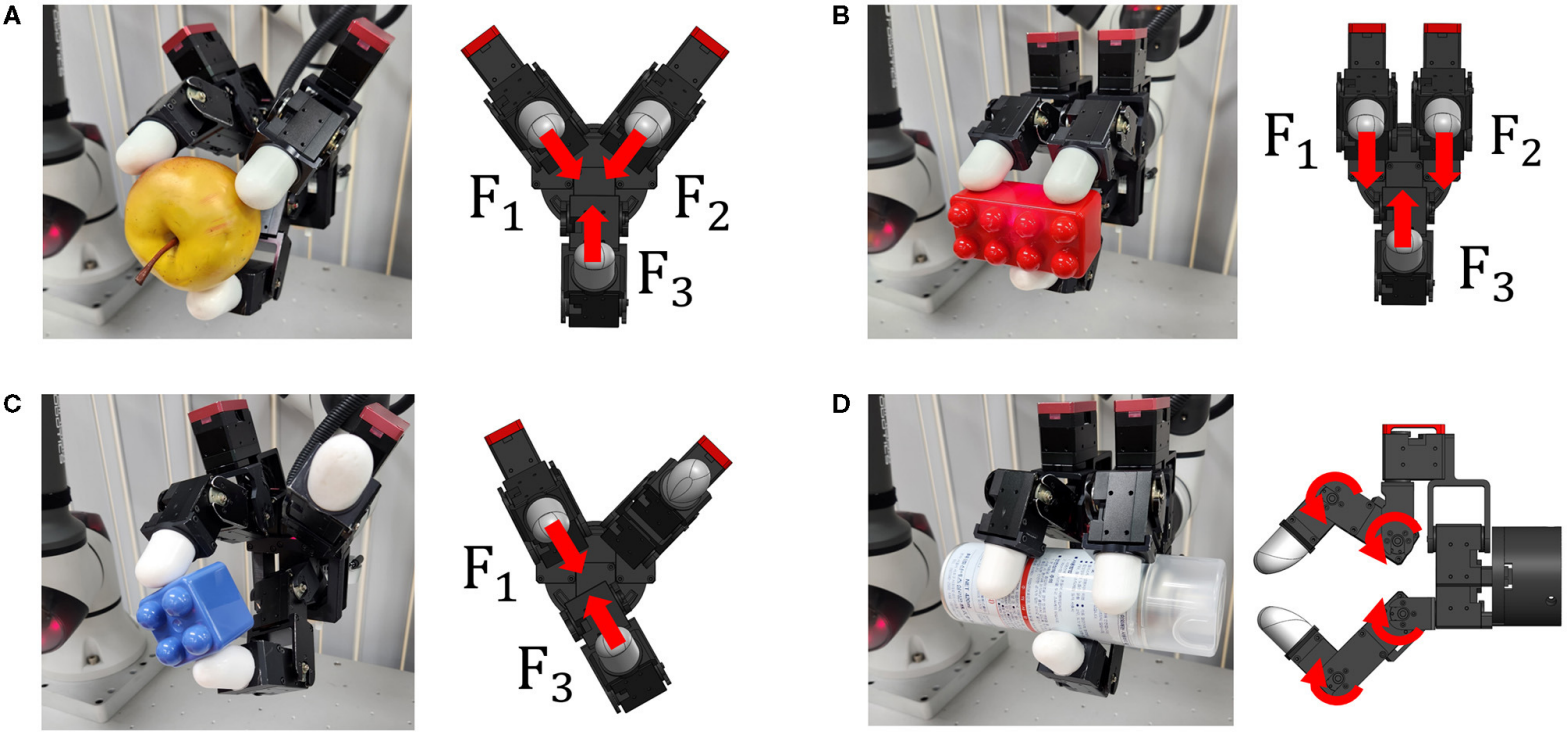
Photograph showing four grasping postures. **(A)** Three-finger grasping; **(B)** Parallel grasping; **(C)** Two-finger pinching; **(D)** Envelope.

To manipulate objects with diverse shapes, we utilized a blind grasping algorithm from a previous study (Bae et al., 2012). The algorithm determines contact forces of each contact point to maintain force equilibrium at the geometric centroid of contact points to achieve stable grasping. In this paper, four grasping postures were employed, namely Three-finger grasping, Parallel grasping, Two-finger pinching, and Envelope, as illustrated in Figure 6. Contact forces were directed toward the central point of three fingers and two fingers in the Three-finger grasping (Figure 6A) and the Two-finger pinching (Figure 6C), respectively. The Parallel grasping posture was suitable for cube shaped objects, as shown in Figure 6B, while the Envelope posture was preferable for cylindrical shapes, as depicted in Figure 6D. These four postures were displayed on the augmented GUI in the substep of selecting grasping posture, as seen in Figure 4B.

**Figure 7:**
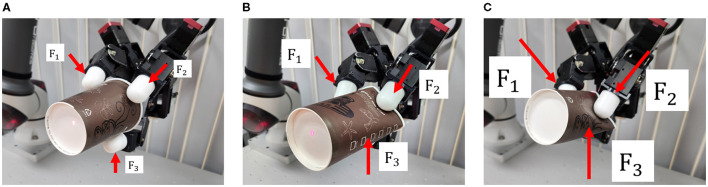
Photograph showing three grip force levels. **(A)** Minimum force (20%); **(B)** Moderate force (50%); **(C)** Maximum force (100%).

The gripper could also adjust the grip force for deformable or delicate objects, which is crucial for preserving their original shape. In this paper, three preset grip force levels were used: Minimum force (20% of Maximum force), Moderate force (50% of Maximum force), and Maximum force. As demonstrated in Figure 7, the paper cup only retained its shape when the minimum grip force was applied (Figure 7A), while stronger grip forces caused deformation, as seen in Figures 7B, C. In the substep of selecting amount of grip force, different levels of the grip force were displayed by using the clockwise arrow (Figure 4D). Blocks near the head and tail of the arrow correspond to the Maximum and Minimum force, respectively, while two intermediate blocks correspond to Moderate force. Human operator involvement is necessary to decide on a proper grasping posture and grip force to grasp undefined objects as this decision is difficult for the robot system.

#### 2.3.3 Shared autonomy control

In the proposed tele-grasping task, the robot manipulator lacked real-time information regarding the workspace environment, including placed objects and destination (designated boxes). Also, it did not use the real-time position of the gripper to reach the object or box. Instead, the human operator had access to this information via the webcam and the laser module, and was responsible for carrying out all substeps of the task. The robot remained stationary until receiving an operator's command, after which it moved the gripper using pre-programmed controls within one second, as long as the operator chose the next gazing block appropriately.

**Figure 8:**
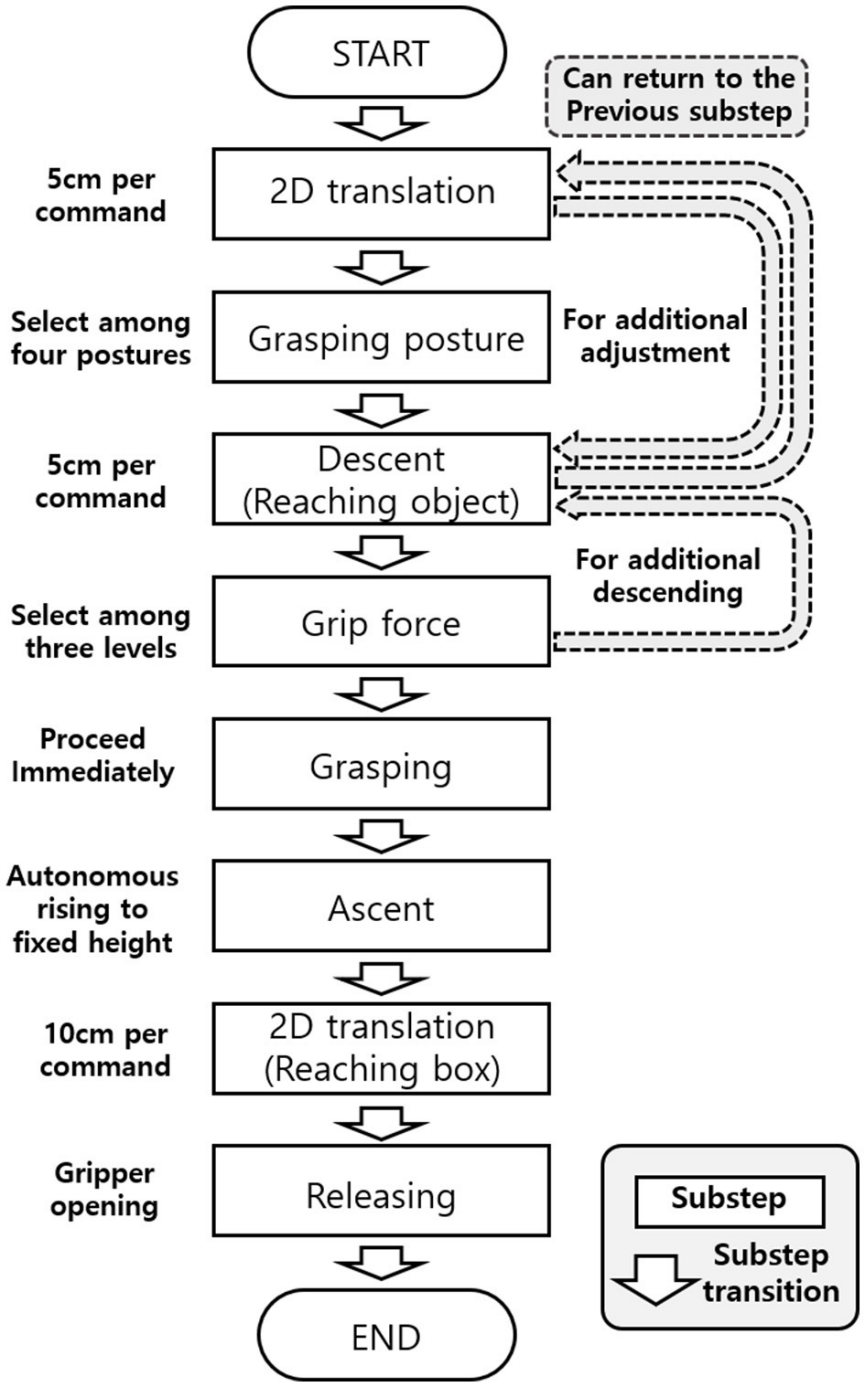
Flowchart for substep transitions.

The splitting of the grasping task into consecutive substeps enabled a simple shared control strategy and increased human involvement. Figure 8 illustrates the flowchart for substep transitions, which involved 2D translation of the gripper, selecting grasping posture, descent to an object, selection of grip force, object grasping, autonomous ascent, reaching a box, and release. By gazing at the designated substep transition block for each substep, the augmented GUI updated the symbolic display to the next substep accordingly. Substep transitions also allowed the operator to return to the previous substep in the gripper's 3D movement, as highlighted by gray dashed arrows in the flowchart, helping the operator reach the object accurately.

The shared control strategy used in the task included not only pre-programmed control but also the blind grasping algorithm that allowed the manipulator to self-adjust during object grasping (Figure 2). To move the gripper, the operator selected one of four directions in the horizontal plane. For grasping, the operator chose one of four postures, three levels of grip force, and the timing of grasping in a sequential procedure. Once the operator had made the high-level decisions necessary for grasping, the manipulator conducted object manipulation with self-adjustment to grasp the object robustly.

### 2.4 Remote data transfer between commander and executor

The command for manipulation of the gripper was transmitted from the commander to the executor, and the real-time video streaming data was transferred from the executor to the commander. The remote data transfer was enabled through a virtual private network (VPN) service provided by a commercial router (R8000, NETGEAR, San Jose, California, USA). Both computers in the commander and the executor were connected to the VPN server of the router as clients via OpenVPN (2.4.7, Pleasanton, California, USA) software.

The command had a string format, as shown in Table 2. Inside the brackets, the alphabetical characters were enumerated in order of operation (O for operation, S for stop), substep (T for translation, G for Grasping, and R for Release), motion (x, y, z for direction to move, and g, p, m, e for each grasping posture), and value (−100 to 100 mm for distance to move, and 1 to 3 for level of grip force). This command was transmitted to the manipulator via the User Datagram Protocol/Internet Protocol (UDP/IP). On the other hand, the webcam data was transferred through a real-time streaming protocol (RTSP) server. The video data was encoded using the High-Efficiency Video Coding (HEVC) format with FFmpeg. An inexpensive webcam (C920, Logitech, Lausanne, Switzerland) with 30 frames per second (fps) and a resolution of 640 × 480 was used to capture the executor robot system.

**Table 2:** Commands for remote data transfer between commander and executor.

**Operation**	**Substep**	**Motion**	**Value**
O	T	x	−100 to 100
		y	−100 to 100
		z	−100 to 100
O	R	g	0
		p	0
		m	0
		e	0
O	G	g	1 to 3
		p	1 to 3
		m	1 to 3
		e	1 to 3
S			

## 3 Experimental validation

To validate the functionality of the system, 10 healthy subjects who were novices to BCI teleoperation were recruited. The subjects were 1 female and 9 males, and their ages were in the range of 22–28. The experiment was conducted in a standard office environment and took less than two hours to complete. The subject was directed to sit on a chair and gaze at the monitor displaying the GUI at a distance of approximately 50 cm. The height and orientation of the chair were adjusted to align the subject's gaze direction with the center of the monitor screen (Figure 1B). The EEG-BCI system was applied by fitting an EEG cap equipped with 11 electrodes, including a ground electrode at the forehead, a reference electrode on the crown, and 9 electrodes in the occipital lobe. The conductive gel was inserted until all impedances were lower than 10 kΩ.

### 3.1 Preliminary calibration

**Figure 9:**
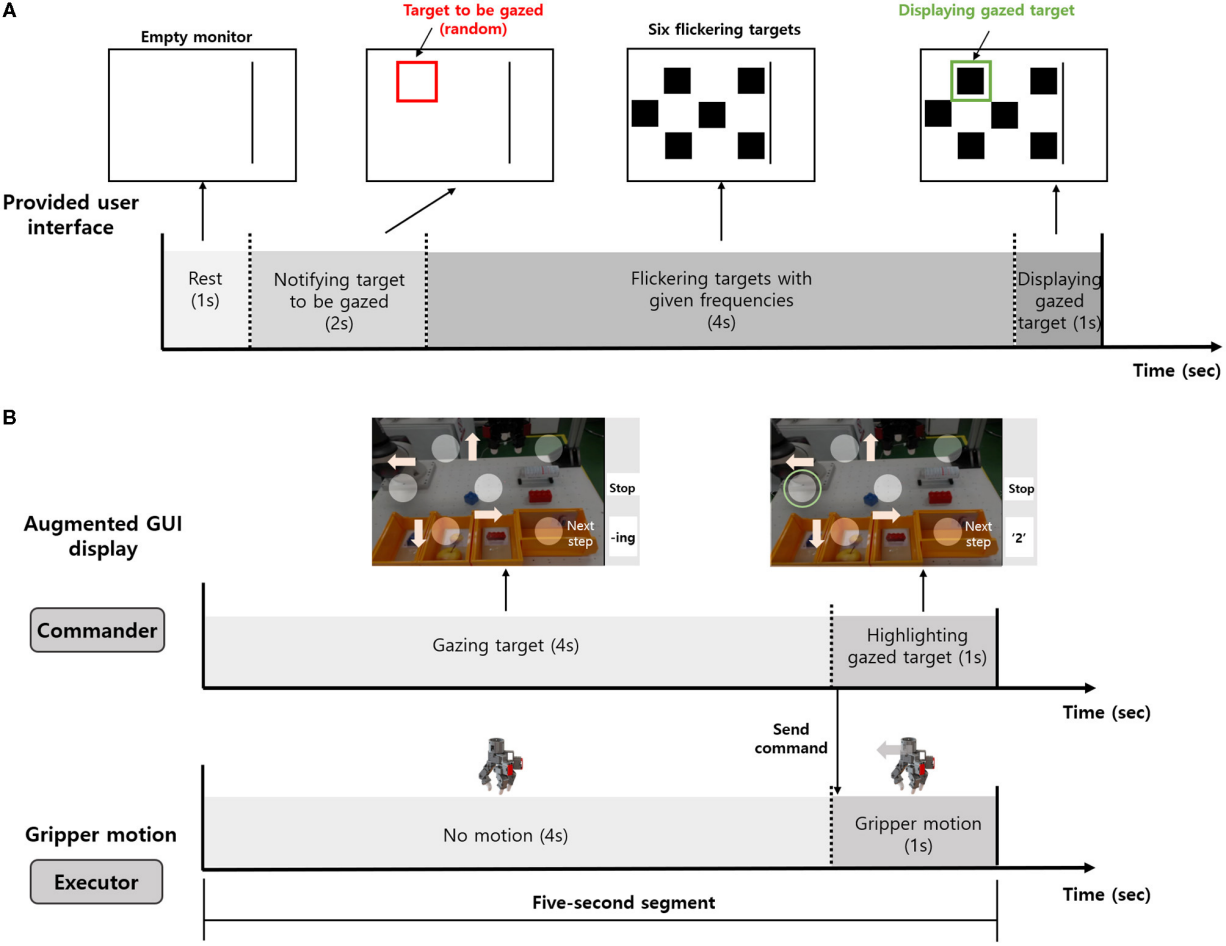
Experimental protocol. **(A)** Preliminary calibration to increase the accuracy of SSVEP. **(B)** Real-time tele-grasping (Five-second segment).

Prior to the tele-grasping task, a preliminary calibration was implemented to check the efficacy of the expectation of the gazed block by using the standard CCA and improve the accuracy by the individual adjustment process. Each trial comprised of 1 s rest, 2 s to notify the target to be gazed at which was randomly selected, 4 s of gazing at the notified target, and a final 1 s cue highlighting the expected target, as depicted in Figure 9A. The placement and assigned frequencies of flickering blocks were matched to that used in the augmented GUI. This trial was repeated 60 times, amounting to 480 s of calibration data collection. The selection of the target was randomized in each trial while ensuring an equal distribution across all six flickering blocks (i.e., 10 times for each block).

For each block, the number of times to be gazed at obtained from the CCA method, *n*_*k*_, was measured. The initial set of the correlation coefficients ρ_*i*_ could be considered as a pre-calculated result of an element-wise product by an initial set of weighting factors ω_*i*_ = [ω_1*i*_, ..., ω_*Ni*_] = [1, ..., 1], as shown in Equation (4):


(4) $$\begin{array}{l} \begin{array}{c} \rho_{i}=\left[\omega_{1i}\rho_{1},...,\omega_{Ni}\rho_{N}\right] \end{array} \end{array}$$

Compared to the true number of assigned times for each block as the target, *n*_*t*_ = 10, any excessive (or insufficient) detection of a particular frequency, which means *n*_*k*_>*n*_*t*_ (or *n*_*k*_<*n*_*t*_), necessitates an adjustment by decreasing (or increasing) the associated correlation coefficient ρ_*k*_ in Equation (2). This adjustment was applied by assigning a weighting factor, determined by the ratio of *n*_*k*_ to *n*_*t*_, to each frequency's corresponding correlation ρ_*k*_, as following Equations (5) and (6):


(5) $$\begin{array}{l} \begin{array}{cc} \omega_{c} & =\left[\omega_{1c},...,\omega_{Nc}\right]=\frac{\sqrt{N}}{n_{t}\sqrt{\frac{1}{n_{1}^{2}}+...+\frac{1}{n_{N}^{2}}}}\left[\frac{1}{n_{1}/n_{t}},...,\frac{1}{n_{N}/n_{t}}\right] \end{array} \end{array}$$

The set of weighting factors in Equation (5) was normalized to make the same norm as the set of the initial weighting factors. The original equation of finding the SSVEP frequency by the CCA method, Equation (3), then was revised by multiplying a weighting factor for each correlation coefficient as follows:


(6) $$\begin{array}{l} f_{gc}=\arg\max\omega_{kc}\rho_{k},k=1,2,...,N, \end{array}$$

where *f*_*gc*_ is the expected gazed frequency with the weighting factor. Equation (6) and the obtained set of weighting factor ω_*c*_ were used in the real-time tele-grasping task. This calibration process allowed for personalized frequency extraction, enabling enhanced performance in the subsequent tele-grasping task.

### 3.2 Real-time tele-grasping task

After calibration, the participant was instructed to perform the real-time tele-grasping task using the augmented GUI. Comprehensive explanations and several practice sessions were provided to familiarize the subject with the GUI and the overall task procedure. Prior to the whole task, the subject was informed to perform each substep first, for the benchmarking to show the functionality of each substep. As shown in the task flowchart in Figure 8, the developed system was composed of each substep according to the common grasping repertoire. Only substeps of handling by the subject were selected to test, and the task success rate and average execution time were measured for each substep. These substeps were 2D movement (Task 1), Selection of grasping posture (Task 2), Approaching and grasping (Task 3), and 2D movement and releasing (Task 4).

For each trial of command, the five-second segment composed of four-second of gazing duration and the one second of the gripper motion was continuously repeated during all tasks. To generate the desired command, the subject had to gaze at the corresponding block for this gazing duration. The EEG-BCI system then immediately applied the CCA to detect the gazed block and sent the corresponding command to the robot manipulator. The robot system performed the command to manipulate the gripper within one second. By observing the motion, the subject could decide the next gazing block for the following command before the beginning of the next trial. For instance, in the 2D translation substep (Figure 9B), the subject intended to grasp the cube, and therefore gazed at the flickering block with a symbol of left arrow for four seconds, and then observed for one second the leftward movement of the gripper. This five-second segment was used to not only send the command within the substep but also to shift to the next substep by updating the GUI. Thus, this simple five-second segment was sufficient to complete the entire task.

Task 1 was designed to be completed if the gripper moved twice at minimum then reached the object's plane position, and the success case was measured when the laser illuminated the center of the object. Task 2 required a single decision for each trial, and when the correct posture was made then it counted as a success case. Task 3 started by matching the horizontal position of the gripper with the object. So the gripper was set to reach the object after lowering three times, and then the subject gazed at the next step block to select the grip force. The success case was if the gripper grasped the object properly without the drop. Task 4 started when the gripper completed its automatic ascent, allowing it to release the object after the gripper moved twice at minimum. The success trial was counted only when the object was put in the placed box. The completion time of each substep was averaged over five successful cases, and the success rate was calculated from the total number of attempts to achieve a minimum of five successes.

After testing each substep, the subject was asked to complete the entire grasping task, which was the sequential process from Task 1 to Task 4. While the previous substep tests benchmarked the system in a routine environment, the whole task was designed to demonstrate the system's ultimate goal of grasping undefined objects in an unstructured environment. So the object type was varied across subjects, as well as the object was randomly placed on a 5 cm grid, considering the gripper's capability of 5 cm move per each command, and the box was randomly placed also. However, for a fair comparison, the minimum number of commands to complete the substep was kept the same. Then, the total completion time from the gripper origin to release the object was measured for each subject in the average of three success cases of placing the object in the box.

The success rate and average completion time for each step were measured per subject and tabulated with the overall average results of all participants in Table 3. The total completion time for the successful grasping was also listed in the last column. Noting that the total completion time included successive substep transitions' time to move the next substep. The average success rates of all subjects from Task 1 to Task 4 were 88.3 ± 9.4, 80.6 ± 15.6, 78.6 ± 11.4, and 72.7 ± 17.2% (mean ± SD), respectively. The average completion time of all subjects for each substep was 19.8 ± 7.5, 5.0, 31.5 ± 8.7, and 37.1 ± 12.6 s (mean ± SD). In addition, the average value of the total completion time including substep transition was 115.3 ± 24.5 s (mean ± SD) over 10 subject's results. The result shows that the average success rates of all subjects were over 70% for all steps, and the average completion time for each step was less than a minute, therefore all subjects could perform the entire grasping task successfully without getting tired or fatigued.

**Table 3:** Experimental results of the success rate and the completion time for each substep task and the total completion time including substep transition.

**Subject**	**Task 1** **(2D movement)**		**Task 2** **(Selection of** **grasping posture)**		**Task 3** **(Approaching and** **grasping)**		**Task 4** **(2D movement** **and releasing)**		**Total completion** **time including** **substep transition**
	**Success** **rate (%)**	**Completion** **time (s)**	**Success** **rate (%)**	**Completion** **time (s)**	**Success** **rate (%)**	**Completion** **time (s)**	**Success** **rate (%)**	**Completion** **time (s)**	
Sub 1	90.0	19.8	100.0	5.0	63.6	28.3	62.5	55.0	98.3
Sub 2	100.0	12.0	80.0	5.0	71.4	24.0	75.0	27.0	93.3
Sub 3	83.3	33.0	70.0	5.0	75.0	30.0	50.0	32.5	85.0
Sub 4	71.4	28.0	63.6	5.0	83.3	29.0	100.0	40.0	145.0
Sub 5	100.0	25.0	90.0	5.0	85.7	31.7	55.6	42.0	150.0
Sub 6	83.3	22.0	60.0	5.0	85.7	21.7	66.7	35.0	130.0
Sub 7	100.0	18.3	90.0	5.0	75.0	27.5	83.3	24.0	111.0
Sub 8	85.7	11.7	62.5	5.0	83.3	30.0	62.5	36.0	101.7
Sub 9	85.7	18.3	90.0	5.0	62.5	51.0	100.0	59.0	143.3
Sub 10	83.3	10.0	100.0	5.0	100.0	42.0	71.4	20.0	95.0
Avg	88.3 ± 9.4	19.8 ± 7.5	80.6 ± 15.6	5.0	78.6 ± 11.4	31.5 ± 8.7	72.7 ± 17.2	37.1 ± 12.6	115.3 ± 24.5

**Figure 10:**
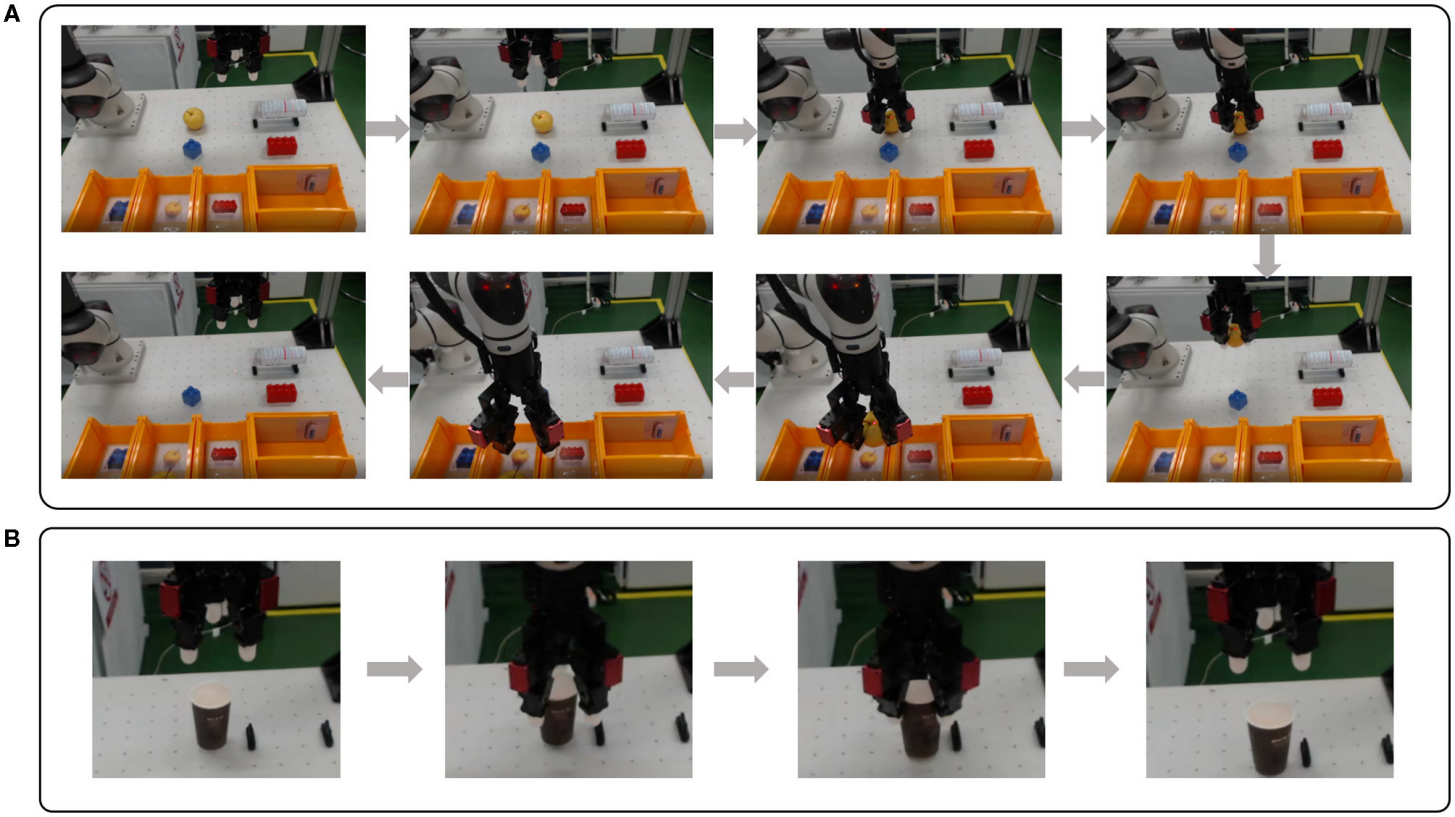
Examples of tele-grasping with proper posture and grip force. **(A)** Snapshots for tele-grasping an apple using Three-finger grasping posture (captured from Supplementary Video). **(B)** Snapshots for tele-grasping a paper cup with minimum grip force (captured from Supplementary Video).

As examples of tele-grasping demonstrations, the task progresses of apple and paper cup were introduced in Figure 10. The order of snapshots aligns with the task flowchart displayed in Figure 8. The apple was picked by Three-finger grasping posture and Maximum grip force, and released in the box with the apple image. Supplementary Video includes not only the whole demonstration of the apple but also parts of tele-grasping of other objects with various grasping postures. The paper cup was prone to crushing when grasped tightly. So, to preserve its shape, the subject selected the minimum level of grip force. A separate demonstration of testing the impact of varying grip force when grasping the paper cup is shown in Supplementary Video.

## 4 Discussion and conclusion

This article introduces a tele-grasping system that employs real-time EEG and an augmented GUI to elicit SSVEP and enable sequential decision-making. The developed system expands the scope of human operator involvement by subdividing the grasping task into multiple substeps and designing an augmented GUI for efficient sequential procedure. The GUI enables the human operator to select various grasping parameters, including the position of the gripper, grasping posture, and amount of grip force, using a reduced number of flickering blocks, not exceeding six. The proposed system is tested in a commander-executor configuration, where the human commander can make decisions for the proper grasping by using the augmented GUI, and the distantly located executor of the robot system performs the received command to move the gripper. Experimental results demonstrate that the proposed system successfully grasps objects of various shapes and deformability through the human operator's sequential decisions.

The developed system, however, exhibits several limitations that affect its performance. First, the system has a minimum 4 s of tele-grasping delay to move the gripper due to the gazing duration for sending commands. During the real-time tele-grasping task, each trial is continuously repeated for every 5 s, where the first 4 s is the gazing duration and the following 1 s is for the gripper motion. The execution time of the CCA method and the round-trip latency for the remote data transfer are short enough to complete the gripper motion within the given one second, enabling the participant to select the next command before the onset of the new trial. In addition, the fixed distance (5 cm) to move restricts the system's capability to approach objects not in multiples of 5 cm, despite the blind grasping algorithm's ability to accommodate grasping at a slightly offset (< 1 cm).

Though this tele-grasping delay is the bottleneck of the task completion time, we have focused more on the usability of the system. Reducing the gazing duration impacts the quality of the SSVEP signals, consequently, the system's ability to detect the peak frequency in the SSVEP will be declined, causing erroneous motions frequently against the human intention. The subject then has to fix this situation in further commands, which is not preferable due to redundant mental load causing fatigue, and disturbing concentration on the gazing. Even though our system sacrificed the task completion time, we placed more importance on decreasing wrong commands to be beneficial to the task success rate and improve usability.

The standard CCA method's relatively low accuracy also adversely affects the system due to the wrong detection of the gazed target. The proposed system tackles this accuracy problem in two ways: Preliminary calibration and reducing decision options. The SSVEP response from the flickering of given frequencies is different from person to person. So individual calibration is necessary to compensate for either underestimated or overestimated frequency. The calibration process determines the set of weighting factors and each weighting factor is multiplied by the corresponding correlation coefficient. The concept of weighting to the correlation coefficient is similar to the previous research (Shi et al., 2019), but the detailed algorithms of determining the weighting factor are different, as well as our system uses this weighting process as a preliminary calibration for improving the real-time teleoperation. Even in the real-time task, if an unusually high detection of a particular frequency suddenly happens from the self-report or the observation, we rebalanced the weights accordingly. Also, subdividing the whole grasping task into sequential substeps reduces the required decision options to six or fewer for each substep. For instance, the 3D movement can be carried out in the order of plane movement and descent to the object, and selecting postures and grip forces can be covered within four commands.

However, these approaches are not sufficient to solve the system limitation of the long completion time. This is because the main source of elongating the completion time is the tele-grasping delay due to the four-second gazing duration as mentioned above. Future research should mainly focus on decreasing tele-grasping delay by implementing an advanced frequency detection algorithm of the SSVEP in a short gazing duration (Chen et al., 2018; Li and Kesavadas, 2022). In addition to applying other methods of SSVEP analysis, to further improve task efficiency, we may consider combining our EEG-controlled system with other methods of decoding EEG signals, or even other hands-free teleoperation control schemes beyond the EEG, such as eye tracking or voice-based control for future studies. For instance, there have been proposed hybrid systems that combine the SSVEP-BCI with motor imagery (MI) (Cao et al., 2021) or eye-tracker in grasping tasks (Guo et al., 2023).

Although these alternative methods would show better system performance, the main purpose of our study is to explore the technology completeness of EEG-based tele-grasping systems, rather than focusing on advancing task efficiency or perfectness. To assist patients with upper limb impairments in conducting ADLs, a hands-free teleoperation control system is required for grasping diverse objects even for undefined to the robot manipulator. Among several hands-free teleoperation methods, our attention is drawn to the observation that existing EEG-based demonstrations of grasping confined the task to pre-defined objects and utilized the object information. The ability of the EEG-controlled tele-grasping system needs to be extended for grasping undefined objects in order to be applied to their everyday life.

To show the capability of grasping undefined objects, we designed the experiment as benchmarking of each substep first and then tested the whole grasping task composed of a sequential combination of all substeps. These substeps of Task 1 to Task 4 are the common repertoire of the grasping tasks. The experimental result shows that every subject succeeded not only in all substeps separately but also in the whole task. Therefore, the benchmarking of each step shows the system's functionality of common grasping repertoire, and the demonstrations of the whole task verify the system's further extended capability of grasping undefined objects in an unstructured environment. The system therefore can be used in the daily life of patients, enough to show the technological advancement in EEG-controlled grasping systems.

Additionally, the developed system has been designed to become a general EEG-based teleoperation framework, making it more practical and useful for potential users of the system. So, the system is also suitable for other teleoperation tasks that involve sequential decision-making by the human operator. The GUI can be used in standard commander-executor teleoperation setups, where the commander receives robot information through video streaming and controls its hardware (Zhao et al., 2017). By dividing tasks into multiple substeps and using symbols to assign appropriate commands to flickering blocks, tasks can be executed remotely. The GUI updates every substep transition, while the blocks remain consistent in shape, frequency, and position, regardless of the substep, simplifying the implementation of every task.

In conclusion, robot grasping of undefined objects in an unstructured environment, which is challenging to the robot system alone, is accomplished by involving real-time human decisions through EEG-controlled teleoperation. These multiple decisions can be facilitated by our augmented GUI, which empowers the human operator to move the gripper in 3D and select a grasping posture and grip force level in a sequential procedure. Tele-grasping for objects with diverse shapes, sizes, and fragilities has been successfully demonstrated. This system can be effectively used in teleoperation tasks where real-time decision-making is necessary.

## Data availability statement

The raw data supporting the conclusions of this article will be made available by the authors, without undue reservation.

## Ethics statement

The studies involving humans were approved by Korea Advanced Institute of Science and Technology Institutional Review Board (KH2021-219, KH2023-168). The studies were conducted in accordance with the local legislation and institutional requirements. The participants provided their written informed consent to participate in this study.

## Author contributions

MK: Conceptualization, Data curation, Formal analysis, Investigation, Methodology, Validation, Writing—original draft, Software. M-SC: Data curation, Methodology, Software, Writing—review & editing. G-RJ: Formal analysis, Software, Writing—review & editing. J-HB: Conceptualization, Supervision, Writing—review & editing. H-SP: Conceptualization, Funding acquisition, Project administration, Resources, Supervision, Writing— review & editing.
